# High-Density Glasses in Blepharospasm: Influence of Migraine and Early Morning Effects

**DOI:** 10.7759/cureus.94664

**Published:** 2025-10-15

**Authors:** Masato Wakakura, Akiko Yamagami

**Affiliations:** 1 Ophthalmology, Inouye Eye Hospital, Tokyo, JPN

**Keywords:** benign essential blepharospasm, early morning effects, high-density glasses, led strain, migraine, non-motor symptoms, photophobia, photosensitivity, quality of life (qol), treatment trial

## Abstract

Photophobia and somatosensory disorders in patients with benign essential blepharospasm (BEB) are caused by the accumulation of light input to the brain, resulting in persistent eye-opening difficulties. Consecutive patients with photophobia and photosensitivity due to BEB were recruited. The patients were asked to participate in a trial using high-density glasses, with only 1.5% visible light transmittance, produced by Tokai Optical Co. Ltd. (HD glass®) (Aichi, Japan). Patients were instructed to wear glasses 2-3 times per day for 20 min or more in a dimly lit room every day for at least two months. Patients answered a questionnaire on the effects of wearing glasses whilst wearing them (during), approximately 30 min after removing the glasses (after), and two months after the self-trial (two-month). A total of 61 patients (52 women and 9 men) aged 21-81 (51.5±17.4) years were recruited. Early morning effects were identified in 39 patients (64.0%), and a present or previous history of migraine was reported in 29 patients (47.5%). More than half of the patients eventually reported improvement. The improvement rate was significantly higher at all three time points (during, after, and two months) in the group with early morning effects than in the group without early morning effects. Migraines, which are often associated with photophobia, may be a risk factor for BEB. This trial, using high-density glasses to inhibit light input to the brain, proved beneficial, particularly in patients with BEB and early morning effects, increasing their quality of life after two months. However, controlled trials are needed to validate these findings.

## Introduction

Benign essential blepharospasms (BEB) are associated with focal dystonia. BEB involves both motor and non-motor features; the latter include sensory symptoms such as eye soreness, pain, and photophobia, as well as psychiatric features such as anxiety disorders, depression [1], and sleep disorders [2]. Photophobia and extreme photosensitivity without eye disease may be mediated by the photophobia circuit, as described by Digre et al., in BEB and migraines [3]. Continuous photophobia substantially decreases vision quality: BEB is a syndrome of persistent eye use difficulty [4].

Several people with BEB have few or no symptoms in the morning, with symptoms worsening throughout the day (this was termed the ‘early morning effect’). This phenomenon may stem from a lack of or minimal light input from the eyes to the brain when in bed or asleep. Thalamic hyperactivity has been reported in patients with photophobia [3-6]. Considering these findings, reducing the light input to the thalamus during the daytime may be a treatment option for BEB.

To test this hypothesis, we conducted a prospective interventional case study using high-density tinted glasses.

## Materials and methods

Consecutive patients with BEB who met the clinical and study criteria were recruited. Patients diagnosed with BEB according to the Japanese clinical guidelines were enrolled. The guidelines were written in Japanese but were in accordance with those applied in a previous study [4]. A diagnostic algorithm was developed based on facial expression and a patient self-reporting checklist (Table 2 in [4], an open-access journal); Table 1 in the appendices and voluntary blinking tests (Table 1b in [4], an open-access journal) and Table 2 in the appendices. Ocular surface diseases (e.g., dry eye, Sjögren's syndrome, and corneal epithelial erosion) that could account for the symptoms of BEB were ruled out at diagnosis using slit-lamp microscopy.

Whether participants with BEB experienced photosensitivity was assessed through clinical interviews and a self-reporting checklist. Patients who responded positively to the following two statements were included: 1) I am sensitive to light when I go outside or even inside a building, and 2) I usually stay indoors because of my aversion to sunshine. These two statements were both deemed indicative of photophobia.

We also recruited patients who found LED lighting intolerable (LED strain) or who experienced intractable motor or non-motor symptoms, including severe eye strain, physical fatigue, ocular or periocular pain/irritation, headache, nausea, and a floating sensation, while viewing screens and displays, including smartphones, tablets, personal computers, and televisions. Some of these individuals were not initially aware that their symptoms were related to light sensitivity; therefore, we referred to this phenomenon as ‘delayed photosensitivity.’

All patients with photophobia or delayed photosensitivity were included in this study. The exclusion criteria were as follows: 1) patients who received botulinum treatment within one month, 2) patients who underwent eyelid surgery within one year, 3) patients who received a new or additional prescription or withdrawal of psychotropics within six months, and 4) patients diagnosed with mental disorders by a psychiatrist. After obtaining informed consent, a clinical interview was conducted to determine the presence or absence of migraines, family history of early morning effects, and treatment history.

**Figure 1:**
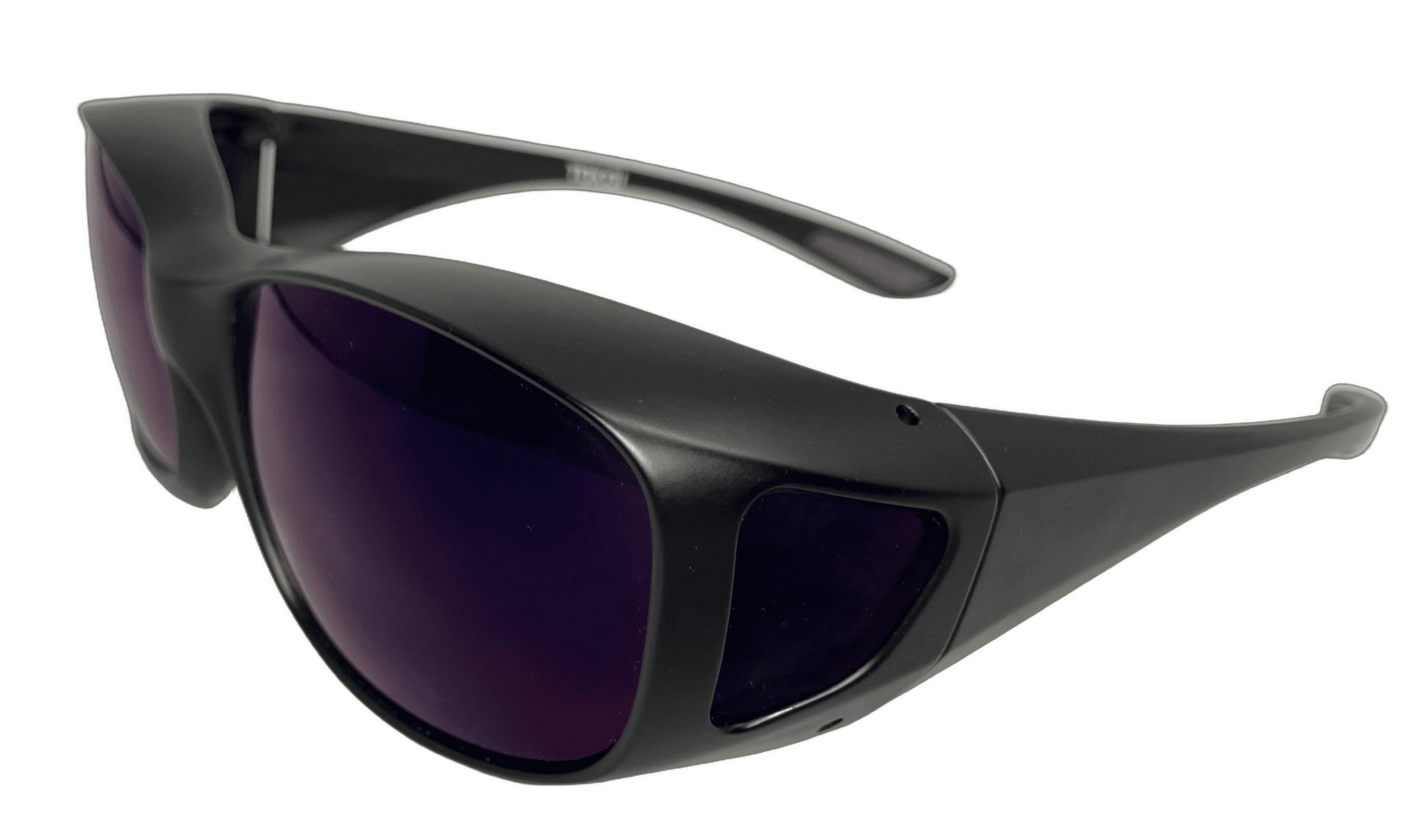
Appearance of high-density glasses (HD glass) used in the study. The photograph is provided with courtesy by Tokai Optical Co., Ltd., Okazaki City, Aichi, Japan.

High-density (HD) glasses with stained, UV-cut tinted lenses (>99.9% UV protection) were used in this trial. A custom-designed lens with only 1.5% visible light transmittance (HD glass, Tokai Optical Co., Ltd., Okazaki City, Aichi, Japan) was manufactured at our request (Figure 1). The reasons for using HD glasses and the guidelines for their use during the trial were as follows: 1) wearing the glasses 2-3 times per day for 20 min or more in a room with dim lighting; 2) not removing the glasses in a brightly lit room or during daytime outdoors during or immediately after the trial; and 3) continuing the above trial daily for at least two months as much as possible.

During treatment using HD glasses, patients were asked to stay relaxed, without necessarily keeping their eyes closed. It was also explained to patients that the authors expected to maximize the natural recovery of their brain disturbance by preventing light input during treatment.

Patients completed a questionnaire survey after a self-trial with HD glasses for 2-3 months, requesting estimates of their eye comfort while wearing the glasses (during), approximately 30 min after removing the glasses (after), and after two months or more (two-month) of wearing the glasses compared with that before the trial. Patients were asked to rate their eye symptoms using the following scale: very good (improved) to good (slightly improved), no change (not improved), and worse at each time point (during, after, and two months). They were also instructed to evaluate their overall eye and brain comfort. These self-assessment scores were rated as 3 (very good), 2 (good), 1 (no change), and 0 (worse). Patients were asked to undergo subsequent clinical examinations after the conclusion of the trial.

Chi-square tests with Yates correction and non-parametric t-tests were used for statistical analysis. Ratings of "very good" and "good" were considered to indicate decreased symptoms in this statistical analysis, whereas "no change" and "worse" were considered to indicate no improvement in symptoms. This study was conducted in accordance with all the relevant national regulations and tenets of the Declaration of Helsinki. The study protocol was approved by the Institutional Review Board (202406-6).

## Results

A total of 52 women and 9 men, ranging in age from 21 to 81 years (mean, 51.5±17.4 years), were recruited. Early morning effects were observed in 39 (64.0%) patients. A current or previous history of migraine was identified in 29 patients (47.5%), a family history of migraine in 24 patients (39.3%), and both in 19 patients (31.1%). All patients declared that they had continued the trial for more than two months for at least five days per week; 30 patients wore glasses once or fewer times per day (low-compliance group), and 31 patients wore glasses twice or more times per day.

**Figure 2:**
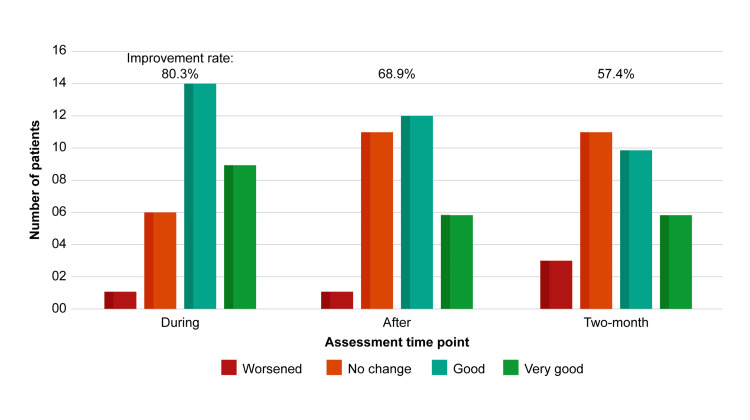
The questionnaire responses for self-assessment are shown at every three time points (n=61). Three points are illustrated on the horizontal axis: the time during the wearing of the high-density glasses (during), 30 minutes after removing the glasses (after), and daytime after two months or more (two-month) of wearing the glasses. The vertical axis indicates the number of patients. Symptom improvement rates were calculated by dividing the total number of ‘good’ and ‘very good’ responses; more than half of the patients rated their symptoms as good or very good at all three points.

Figure 2 shows that more than half of the patients rated their symptoms as good or very good, and the proportion reporting improvement in symptoms gradually decreased over time: 80.3%, 68.9%, and 57.4% at the during, after, and two-month time points, respectively.

The average, standard deviation, and 95% confidence intervals of self-rating are as follows: 2.00±0.88, 1.78~2.22 (during); 1.89±0.73, 1.70~2.06 (after); and 1.66±0.85, 1.44~1.87 (two-month), respectively.

**Figure 3:**
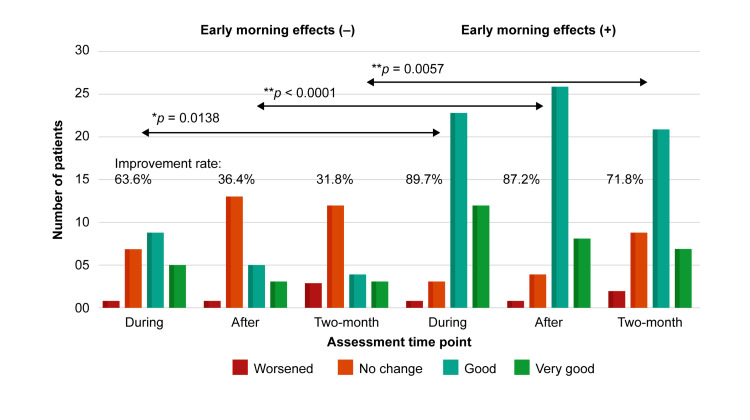
Comparison of questionnaire responses at each of the three time points (during, after, and two-month; see Figure 2) between patients with (n=39) and without (n=22) early morning effects. Symptom improvement rates were calculated by dividing the total number of ‘good’ and ‘very good’ responses by 22 (early morning effects ) and 39 (+), respectively. p<0.05, * p<0.01 according to the chi-square test with the Yates correction. The symptom improvement rate in the group with early morning effects was significantly higher at all three time points. Symptom improvement rates were calculated by dividing the total number of ‘good’ and ‘very good’ responses by 22 (early morning effects ) and 39 (+), respectively. p<0.05, * p<0.01 according to the chi-square test with the Yates correction. The symptom improvement rate in the group with early morning effects was significantly higher at all three time points: during, after, and two months; please refer to the legend of Figure 2 regarding each time point.

The symptom improvement rate in the group with early morning effects was >80% at the during and after time points (63.6%: 89.7%, p=0.0138 during; 36.4%: 87.2% after, p<0.0001; Figure 3). The symptom improvement rate remained high at the two-month time point, with 71.8% of patients with early morning effects and 31.8% of those without early morning effects reporting improvement (p=0.0057).

The statistical results of self-rating at three time points are provided in Table 1. The difference of average rates was statistically indicated between the presence or absence of early morning effects (p <0.05).

**Table 1:** The results of self-rating by patients at three stages are indicated for each characteristic. 1) average±standard deviation, 2) 95% confidence interval. n= number of patients analyzed statistically. P-value is calculated using a non-parametric t-test.

Early morning effects		(+)	(-)	p-value (t test)
	During	2.21±0.70（n=39)^1)^	1.73±0.98（n=22)	p＝0.02
		1.97~2.39^2)^	1.83~2.54	
	After	2.03±0.63	1.62±0.86	p=0.04
		1.83~2.22	1.26~1.98	
	Two-month	2.05±0.64	1.45±0.80	p=0.002
		1.85~2.28	1.11~1.79	
Migraine		(+)	(-)	
	During	1.86±0.88(n=29)	2.13±0.87(n=32)	p=0.003
		1.54~2.18	1.82~2.46	
	After	1.66±0.72	2.03±0.74	p<0.0001
		1.39~1.92	1.78~2.29	
	Two=month	1.69±0.85	1.63±0.82	p=0.77
		1.38~2.00	1.31~1.94	
High Compliance		(high compliance)	(low compliance)	
	During	1.97±0.95(n=31)	2.03±0.81(n=30)	p=0.77
		1.64~2.30	1.74~2.32	
	After	1.89±0.67	1.83±0.83	p=0.85
		1.65~2.12	1.53~2.13	
	Two-month	1.68±0.79	1.63±0.93	p=0.84
		1.09～2.27	1.30～1.97	

The symptom improvement rates at the during, after, and two-month time points were 84.4%, 65.6%, and 50.0%, respectively, in patients without migraine (n=32) and 75.9%, 72.4%, and 65.5%, respectively, in patients with migraine. The results at all three time points did not significantly differ between patients with and without migraine (p=0.6681, 0.2768, and 0.3347, respectively). The patients’ ratings did not differ significantly between patients with or without migraines or with or without a family history of migraines. However, as shown in Table 1, the difference of average rates was statistically indicated between the presence or absence of migraine history only during and after (p<0.01).

**Figure 4:**
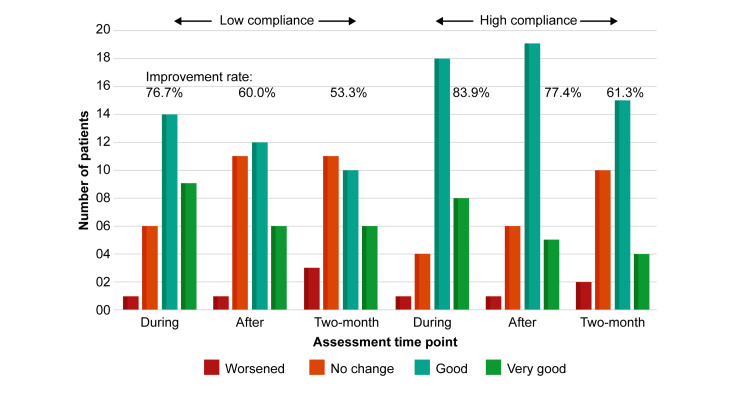
Comparison of questionnaire responses at each of the three time points (during, after, and two-month; see Figure 2) between patients in the low-compliance (n=30) and high-compliance (n=31) groups. Symptom improvement rates were calculated by dividing the total number of ‘good’ and ‘very good’ responses by 30 and 31 in the low- and high-compliance groups, respectively. The symptom improvement rates did not significantly differ at any of the three time points between the groups, as assessed using the chi-square test with Yates correction.

Adverse effects were reported in three cases: fear caused by darkness while wearing glasses in two patients and additional ocular irritation in one patient. However, these three patients continued to participate in the trial. A comparison between the low- and high-compliance groups revealed that although the glasses appeared to be more effective in the high-compliance group, the groups did not significantly differ at the during (76.7%, 83.9%, p=0.6999), after (60.0%: 77.4%, p=0.2332), or two-month (53.3%, 61.3%, p=0.7119) time points (Figure 4).

## Discussion

In our clinical experience, most patients with blepharospasm have photophobia that responds immediately to any light. These patients commonly experience LED strain or intolerance to prolonged viewing of screens that emit light, such as PCs, tablets, smartphones, and televisions. The latter type of intolerance is termed ‘delayed photosensitivity’ and, in severe cases, “extreme photosensitivity.” In the present study, we included both types of light intolerance: photophobia and photosensitivity. Delayed photosensitivity, or the accumulation of light input from the eyes to the brain, may induce non-motor symptoms, including photophobia, soreness, pain, dry eye sensation, eye strain, headache, and other somatic and psychiatric symptoms [4].

Chromatic lenses have previously been used in patients with BEB and photophobia [7,8]. The severity of photophobia can be assessed and modulated using such lenses, which may also help with the management of BEB symptoms. While chromatic lenses are typically worn in daily life, the HD glasses used in this study were applied exclusively for therapeutic purposes at specific time intervals during the day. The primary reason for using glasses in this trial was to block light stimulation. More than 60% of all patients and 85% of patients with early morning effects felt comfortable both during and after wearing the HD glasses. These high comfort rates contributed to the continuation of the trial for more than two months. At the two-month time point, 57.4% of all patients and 71.8% of patients with early morning effects felt comfortable with the glasses and reported improved quality of life. These results were not only due to the transient effects of light inhibition but also the proactive effects on brain mechanisms through the photophobia circuit. Early morning effects reflect the activity of the photophobia circuit at night. The repeated circuit rest during the day could improve treatment results with HD glasses, possibly facilitating a spontaneous recovery process. Currently, BEB is difficult to treat, with botulinum toxin treatment being the only therapy available. Therefore, treatment with HD glasses is a viable option for patients undergoing BEB, especially those experiencing early morning effects. However, this study was not randomized or controlled, which represents a limitation.

A high migraine rate (47.5%) was identified in patients with BEB. Migraine is commonly associated with visual sensory disorders, and extreme photosensitivity is a common symptom [3,9,10]. The prevalence of migraine is 3.8%-8.4% in Japan [11,12], which is approximately half of that in Western populations. Digre et al. [3] listed migraines and BEB as causes of photophobia in individuals without eye disease; however, they failed to identify a relationship between migraines and BEB. This is likely because migraines are common among people in Western countries; hence, their association with BEB may not be easily noticed. However, this study found a significant association between migraine and BEB in the Japanese population. It is well known that patients with both BEB and migraine are predominantly female. These characteristics suggest that sensory hypersensitivity in migraine may predispose individuals to the development of BEB. Thus, migraine should be considered as a background risk factor for BEB.

We expect high compliance use of HD glasses to be effective. The effects of HD glasses appeared to be strongest in patients in the high-compliance group; however, the difference was not statistically significant compared to the low-compliance group. This was likely because patients varied in the severity and quantity of their photosensitivity; however, the sample size was too small to conduct a subgroup analysis.

The difficult quantification of photosensitivity was another limitation. As described previously, this study is limited by its non-randomized and uncontrolled design. An important consideration is the difficulty of standardizing assessment scales for symptoms related to high photosensitivity, such as the VAS scale, given the complex nature of these symptoms, which are not solely dependent on light intensity. These limitations may have caused the occurrence of type I/II errors.

Furthermore, this study was not randomized or controlled, and the small number of subjects for subgroup analysis is a significant limitation. Additionally, some data was based on verbal self-reporting and could not be confirmed in an objective manner. As this study was only subjectively assessed, we are planning to conduct an electrophysiological study using electroencephalography in the future.

High photosensitivity appears to be the core symptom of BEB, which significantly impacts quality of life [4]. Nevertheless, there is no beneficial treatment available. Although this study is essentially an uncontrolled, prospective case series, the relatively favorable results, including almost complete disappearance of excessive photosensitivity in four patients, encouraged us to recommend using HD glasses for subsequent patients with early morning effects at our clinic, which treats more than 50 patients.

We expect that the use of HD glasses may provide repeated rest for photosensitivity-related brain circuits; however, it cannot be guaranteed that this would have a direct effect on the brain. Thus, clarification of the pathomechanism underlying photosensitivity is necessary to identify the correct treatment for patients with extreme photosensitivity in both BEB and migraine, as the underlying mechanisms may be linked.

## Conclusions

In conclusion, the present study found that HD glasses showed promise in improving comfort and quality of life in patients with BEB, particularly those with early morning effects; however, controlled trials are needed to validate these findings.

## Appendices

**Table 2:** Blepharospasm self-report check list

Please read the sentences below and check yes (Y) or no (N)		
1	I blink a lot. Y N	
2	I am sensitive to light when I go outside, or even when staying inside a building. Y N	
3	I cannot keep my eyes open. (I prefer to keep my eyes closed.) Y N	
4	I am always annoyed about my eyes: feeling dry, bleary, irritation or pain. Y N	
5	I often bump into other people when I walk in a crowd. Y N	
6	I have bumped into a parked car, a post, or a tree on the street. Y N	
7	I usually stay indoors because of aversion to sunshine, wind, or stairs. Y N	
8	I discontinued driving and/or cycling to be safe. Y N	
9	I sometimes need to use my hands to open my eyes. Y N	
10	I tend to close one of my eyes. Y N	

**Table 3:** Voluntary blinking tests

Please perform the following actions and choose one of the four options that best describes your performance. Numbers 1 and 2 are indicative of abnormal blinking, while number 3 is indicative of the inability to blink.	
Test A. Blink quickly and lightly (approximately 70 times per minute) without moving your eyebrows.	
0	I can do it.
1	My eyebrows move; I blink only tightly.
2	I can’t blink; I can’t help closing my eyes when I try to blink.
Test B. Blink for 10 seconds as quickly and lightly as possible.	
0	I can blink lightly more than 30 times in 10 seconds in good rhythm.
1	I can blink lightly about 30 times in 10 seconds with interruptions.
2	I can’t blink lightly in good rhythm; I sometimes blink tightly.
3	I can’t blink quickly and lightly
Test C. Close your eyes tightly and then open them quickly. Repeat the action 10 times.	
0	I can do it.
1	I can’t open my eyes quickly several times and/or experience difficulty trying to open my eyes quickly.
2	I can’t open my eyes quickly several times and/or experience difficulty trying to open my eyes quickly.
3	It is extremely difficult to open my eyes; or I can’t perform the action 10 times.

## References

[1] Scorr LM, Cho HJ, Kilic-Berkmen G (2022). Clinical features and evolution of blepharospasm: a multicenter international cohort and systematic literature review. Dystonia.

[2] Yang J, Zhang L, Hou Y (2021). Sex related differences in nonmotor symptoms of patients with idiopathic blepharospasm. Sci Rep.

[3] Digre KB, Brennan KC (2012). Shedding light on photophobia. J Neuroophthalmol.

[4] Wakakura M, Yamagami A, Iwasa M (2018). Blepharospasm in Japan: a clinical observational study from a large referral hospital in Tokyo. Neuroophthalmology.

[5] Emoto H, Suzuki Y, Wakakura M (2010). Photophobia in essential blepharospasm--a positron emission tomographic study. Mov Disord.

[6] McCann JD, Gauthier M, Morschbacher R (1999). A novel mechanism for benign essential blepharospasm. Ophthalmic Plast Reconstr Surg.

[7] Herz NL, Yen MT (2005). Modulation of sensory photophobia in essential blepharospasm with chromatic lenses. Ophthalmology.

[8] Blackburn MK, Lamb RD, Digre KB (2009). FL-41 tint improves blink frequency, light sensitivity, and functional limitations in patients with benign essential blepharospasm. Ophthalmology.

[9] Dolati S, Rikhtegar R, Mehdizadeh A, Yousefi M (2020). The role of magnesium in pathophysiology and migraine treatment. Biol Trace Elem Res.

[10] Wilkins AJ, Haigh SM, Mahroo OA, Plant GT (2021). Photophobia in migraine: A symptom cluster?. Cephalalgia.

[11] Sakai F, Hirata K, Igarashi H (2022). A study to investigate the prevalence of headache disorders and migraine among people registered in a health insurance association in Japan. J Headache Pain.

[12] Sakai F, Igarashi H (1997). Prevalence of migraine in Japan: a nationwide survey. Cephalalgia.

